# Supplementary material for: Measurement of atomic scattering factors by cryoelectron microscopy
Source: Proc Natl Acad Sci U S A. 2026 May 8;123(19):e2528758123. doi: 10.1073/pnas.2528758123 (PMC13167779; doi:10.1073/pnas.2528758123)
Supplement: Supplementary file 1 — Appendix 01 (PDF) [file pnas.2528758123.sapp.pdf]

Supporting Information for

Measurement of atomic scattering factors by cryo-electron microscopy

Alexander Shtyrov, Hugh Wilson, Daria Slowik, Keitaro Yamashita, Jade Li, Marcin Wojdyr, Shaoxia Chen, Greg McMullan, Jude Short, Christopher J. Russo, Richard Henderson and Garib N. Murshudov

Garib N. Murshudov
E-mail: garib@mrc-lmb.cam.ac.uk
Richard Henderson
E-mail: rh15@mrc-lmb.cam.ac.uk

This PDF file includes:

Supporting text
Figs. S1 to S3
Tables S1 to S2
SI References

Supporting Information Text

Background on scattering model. Under the weak phase approximation, the scattering amplitude of an object $F^{(c)}(\mathbf{s})$ is proportional to the Fourier transform of the scattering potential. For electron scattering, this potential is the ESP $V(\mathbf{x})$ (1):

$$F^{(c)}(\mathbf{s}) = -\frac{2\pi}{\lambda^2 E}\frac{E_0 + E}{2E_0 + E}\int_{\mathbb{R}^3} V(\mathbf{x})e^{-2\pi\iota\mathbf{s}^\mathsf{T}\mathbf{x}}\,\mathrm{d}\mathbf{x}\,, \tag{S1}$$

where E_0 is the rest energy of an electron, E is the energy of the accelerated electron, and λ is the electron wavelength. In order to make the calculation of scattering amplitudes tractable for macromolecules, further assumptions must be made about V. One common approximation, which is adopted in this work, is that V can be written as a sum of atomic contributions, that is

$$V(\mathbf{x}) = \sum_{n=1}^{N_{\text{at}}} c_n[V_n * G_n](\mathbf{x} - \mathbf{x}_n), \tag{S2}$$

where V_n is the contribution to the ESP of atom n, $\mathbf{x}_n$ is a 3-vector containing the Cartesian coordinates of the atom, and c_n is the site occupancy. Equation S2 is known as the independent atom model (IAM). The function $G_n(\mathbf{x}) = \frac{8\pi^{3/2}}{\sqrt{\det \mathbf{B}_n}}e^{-4\pi^2\mathbf{x}^\mathsf{T}\mathbf{B}_n^{-1}\mathbf{x}}$ represents the contribution of the ADP of the nth atom $\mathbf{B}_n$, which models the uncertainty in the atomic position. Here $\mathbf{B}_n$ is a positive definite 3×3 matrix. V_n is further assumed to have spherical symmetry:

$$V_n(\mathbf{x} - \mathbf{x}_n) = V_n(|\mathbf{x} - \mathbf{x}_n|) = V_n(r). \tag{S3}$$

Combining Equations S2 and S3 with the weak phase approximation, $F^{(c)}(\mathbf{s})$ may be written (2)

$$F^{(c)}(\mathbf{s}) = \sum_{n=1}^{N_{\text{at}}} c_n f_n(s)e^{-2\pi\iota\mathbf{s}^\mathsf{T}\mathbf{x}_n}e^{-\mathbf{s}^\mathsf{T}\mathbf{B}_n\mathbf{s}/4} \tag{S4}$$

where $s = |\mathbf{s}|$ and $f_n(s)$ is the atomic scattering factor for atom n.

Form of the covariance function. In this work, two assumptions are made to construct a tractable covariance $\Sigma_{ii'jj'}$. Firstly, it is assumed that the covariance factorises into 'frequency' and 'atom type' terms, and scattering factors for different atom types are independent, that is $\Sigma_{ii'jj'} = \delta_{ii'}\Sigma_{jj'}$. The second assumption is that a scattering factor $f(s)$ may be expressed as a non-stationary convolution of a Gaussian white noise process $\phi(b)$,

$$f(s) = \int_0^\infty \phi(b)e^{-bs^2}u(b)\,\mathrm{d}b\,, \tag{S5}$$

where $u(b)$ is the scale of the noise process. Then

$$\begin{aligned} \mathbb{E}\left[f(s)\right] &= 0 \\ \operatorname{cov}\left[f(s), f(s')\right] &= \int_0^\infty\int_0^\infty \mathbb{E}\left[\phi(b)\phi(b')\right]e^{-bs^2}e^{-b's'^2}u(b)u(b')\,\mathrm{d}b\,\mathrm{d}b' \\ &= \int_0^\infty e^{-bs^2}e^{-bs'^2}u^2(b)\,\mathrm{d}b \end{aligned} \tag{S6}$$

Further supposing that $u^2(b)/K = \frac{1}{\beta}e^{-b/\beta}$ is the PDF of an exponential distribution, where K is a normalising constant, the covariance becomes

$$\operatorname{cov}\left[f(s), f(s')\right] = K\left(1 + \beta(s^2 + s'^2)\right)^{-1}. \tag{S7}$$

This construction may be justified as a continuous extension of a discrete summation of Gaussians, the latter being the standard parametrisation for X-ray and electron scattering factors used in macromolecular crystallography (2, 3). For the discretised observation model, $\Sigma_{jj'} = \operatorname{cov}\left[f(s_j), f(s_{j'})\right]$, where $s_j = \frac{d_j + d_{j+1}}{2}$. The main features of functions drawn from our prior is that they are smooth, vanish at $s = \infty$ and have zero derivative at $s = 0$. The latter imposes that the ESP behaves as $O(1/r^2)$ as $r \to \infty$, so does not allow point charge-like behavior (which is impossible in any real chemical system)

Hyperparameter estimation. The noise variance $S_{kk'}$ is estimated in frequency bins using maximum likelihood (ML), as in *REFMAC* (4) and *Servalcat* (5). The ML estimator has a closed form. The noise variance σ_j in $\mathcal{B}_j$ is

$$\sigma_j = \frac{1}{N_j}\sum_{k:\mathbf{s}_k\in\mathcal{B}_j}\left|F_k^{(o)} - F_k^{(c)}\right|^2, \tag{S8}$$

where N_j is the number of coefficients in $\mathcal{B}_j$ and $F_k^{(c)}$ is calculated using tabulated scattering factors. Then

$$S_{kk'} = \delta_{kk'}\sum_{j=1}^{N_{\text{bin}}} 1_{\mathcal{B}_j}(\mathbf{s}_k)\sigma_j, \tag{S9}$$

where $1_{\mathcal{B}_j}$ is the indicator function of $\mathcal{B}_j$ and N_{bin} is the number of frequency bins.

The parameters of the covariance $\theta = \{K, \beta\}$ are estimated by maximising the marginal likelihood of the observations given the parameters, where marginalisation is over values of the scattering factors. This method is an application of the evidence approximation (6). For the model adopted in this work, the negative marginal log-likelihood is

$$\begin{aligned} -\log p(\mathbf{F}^{(o)} \mid \theta) = &\sum_{ii'jj'kk'} F_k^{(o)} S_{kk'}^{-1} F_{k'}^{(o)} - v_{ij} \left(\frac{1}{2}\Sigma_{ii'jj'}^{-1} + M_{ii'jj'}\right) v_{i'j'} \\ &+ \frac{1}{2}\log\det\left(\frac{1}{2}\mathbf{\Sigma}^{-1} + \mathbf{M}\right) + \frac{1}{2}\log\det\left(\mathbf{\Sigma}\right) + \log\det\left(\mathbf{S}\right) + \text{const.} \end{aligned} \qquad \text{[S10]}$$

The inference algorithm first finds the hyperparameters θ that minimise the negative marginal log-likelihood in Equation S10 using the iterative optimiser L-BFGS (7). It then computes the posterior expectation μ_{ij} from Equation 3, which is the solution to a linear system. In order to speed up the hyperparameter estimation, the quantities $M_{ii'jj'}$ and v_{ij} are precomputed.

Inference from multiple cryo-EM maps. The probabilistic model can be extended to datasets containing multiple cryo-EM maps. Maps are assumed to be conditionally independent given the corresponding atomic models. Indexing the map by l, the prior remains unchanged while the likelihood becomes

$$F_{kl}^{(o)} \mid \mathbf{f} \sim \mathcal{CN}\left(\sum_{ij} f_{ij} R_{ijkl}, S_{kk'll'}\right), \qquad \text{[S11]}$$

where $S_{kk'll'}$ is diagonal. The latter property follows from the conditional independence assumption. On redefining $M_{ii'jj'} = \sum_{kk'll'} R_{ijkl}^{*} S_{kk'll'}^{-1} R_{i'j'k'l'}$ and $v_{ij} = \sum_{kk'll'} R_{ijk}^{*} S_{kk'll'}^{-1} F_{k'l'}^{(o)}$, Equation 3 still holds.

To ensure consistency between datasets, $F^{(o)}$ is scaled to $F^{(c)}$ isotropically in frequency bins. The scaling parameter is estimated by ML, as in *REFMAC* and *Servalcat*. The ML estimator for the multiplicative factor D_j that scales $F^{(c)}$ onto $F^{(o)}$ is

$$D_j = \frac{\sum_{k:\mathbf{s}_k \in \mathcal{B}_j} F_k^{(o)} F_k^{(c)*}}{\sum_{k:\mathbf{s}_k \in \mathcal{B}_j} \left|F_k^{(c)}\right|^2}, \qquad \text{[S12]}$$

where $F_k^{(c)}$ is again calculated using tabulated scattering factors.

Robust inference. The scattering model adopted in this work does not precisely describe the data-generating process, since it makes a number of simplifying assumptions (sphericity of atoms, absence of a solvent contribution, and so on). The model is therefore misspecified. Inference is made more robust to this misspecification by using a 'power likelihood' (8), which is constructed by multiplying $M_{ii'jj'}$ and v_{ij} by a constant $\alpha < 1$. The value of α is estimated by a validation set method. For a given value of α, the hyperparameters and scattering factors are estimated using only coefficients with frequencies above 1/5 Å^{-1}. The estimated scattering factors are then used to compute values of $F^{(c)}$ for the held-out frequencies. The value of α maximising the correlation between the calculated and observed coefficients is found by performing a line search.

The strength of signal due to electron scattering varies across a cryo-EM map (9). In any given map, some atoms will therefore be poorly resolved. In order to reduce the influence of such atoms on the inference algorithm, they are not used for estimation of the scattering factors. Atoms with an ADP greater than 1.5 times the interquartile range above the median ADP of atoms in the same structure are considered outliers. The threshold translated to exclusion of 12.9% of atoms in the catalase dataset and 13.4% of atoms in the EMDB dataset. For the catalase dataset, the ADP cutoff values were 92.6 Å^2 for human erythrocyte catalase, 77.9 Å^2 (*Micrococcus luteus*) and 86.9 Å^2 (*Rhizobium radiobacter*). The scattering contribution of excluded atoms is calculated using tabulated scattering factors and subtracted from the map before estimation.

Preparation of datasets. Simulated maps were generated from eight experimental structures of catalase enzymes (PDB depositions 8EL9, 8PVD, 8SGV, 8WZH, 8WZJ, 8WZK, 8WZM, 9BDJ). Ligands, waters and hydrogen atoms were removed and isotropic ADPs were randomised before simulation by drawing their values from an inverse-gamma distribution (10). *GEMMI* and tabulated scattering factors (3) were used to generate ESP maps. Gaussian noise was added to the simulated maps in Fourier space. The power spectrum of the noise was chosen to (a) ensure that the FSC at 1/2 Å^{-1} was equal to 0.143 and (b) produce sigmoidal FSC curves that are typical of high-resolution cryo-EM reconstructions.

The EMDB dataset is a subset of maps deposited in the EMDB with (a) reported resolution of 2 Å or better, (b) an associated PDB deposition with no missing link records and (c) deposited half-maps. Model refinement was performed for each of the maps in the dataset using *Servalcat*. Maps with an average map-model FSC after refinement of below 0.6 were excluded, these were found to have large unmodelled or flexible regions. The box size of each map was then trimmed to within 20 Å of atoms in the model using *Servalcat*. Maps that had more than 300^3 voxels after trimming were excluded. The final dataset consisted of 64 maps, which were randomly split into training (52 maps) and test (12 maps) datasets.

Details of the test set are given in Table S2. The following EMDB entries were used in the training set: EMD-60655, EMD-21024, EMD-29357, EMD-42164, EMD-51522, EMD-28259, EMD-15312, EMD-11657, EMD-26756, EMD-31910, EMD-17508,

EMD-11638, EMD-45626, EMD-45628, EMD-23749, EMD-45192, EMD-16783, EMD-16788, EMD-17522, EMD-27316, EMD-25201, EMD-28759, EMD-26996, EMD-16785, EMD-11233, EMD-16786, EMD-27285, EMD-41150, EMD-17129, EMD-16787, EMD-26801, EMD-19938, EMD-19881, EMD-17521, EMD-61728, EMD-17510, EMD-16789, EMD-42775, EMD-60391, EMD-28812, EMD-16531, EMD-16784, EMD-42793, EMD-44299, EMD-21951, EMD-51230, EMD-61727, EMD-14332, EMD-27286, EMD-17511, EMD-41149, EMD-26757. The EMDB training set contains entries with reported fluences ranging from 27 eÅ^{-2} to 90 eÅ^{-2}. 1 eÅ^{-2} corresponds to a dose of 3.7 MGy (11).

Table S1. Non-hydrogen atom types found in the peptide backbone and amino acid side chains

**amino acid**	**atom types**
backbone	C(CNO), C(HCCN) (C(HHCN) in Gly), N(HCC) (N(CCC) in Pro), O(C)
Ala	C(HHHC)
Pro	C(HHCC), C(HHCN)
Val	C(HHHC), C(HCCC)
Leu	C(HHHC), C(HHCC), C(HCCC)
Ile	C(HHHC), C(HHCC), C(HCCC)
Met	C(HHCC), C(HHCS), C(HHHS), S(CC)
Trp	C(HHCC), C(HCC), C(HCN), C(CCC), C(CCN), N(HCC)
Phe	C(HHCC), C(HCC), C(CCC)
Ser	C(HHCO), O(HC)
Thr	C(HHHC), C(HCCO), O(HC)
Tyr	C(HHCC), C(HCC), C(CCC), C(CCO), O(HC)
Asn	C(HHCC), C(CNO), O(C, amide), N(HHC)
Gln	C(HHCC), C(CNO), O(C, amide), N(HHC)
Cys	C(HHCS), S(HC) (S(CS) in disulphide)
Lys	C(HHCC), C(HHCN), N(HHHC)
Arg	C(HHCC), C(HHCN), C(NNN), N(HHC), N(HCC)
His	C(HHCC), C(HCN), C(CCN), C(HNN), N(HCC), N(CC)
Asp	C(HHCC), C(COO), O(C, carboxylate)
Glu	C(HHCC), C(COO), O(C, carboxylate)

Table S2. Details of structures in the test set

EMDB ID	reported resolution (Å)	fluence (e $Å^{-2}$)	description
EMD-10101	1.84	30.0	apoferritin from mouse
EMD-13937	1.9	45.0	human connexin 26 dodecamer at 90mmHg PCO2, pH7.4
EMD-61729	1.83	50.0	ferritin variant R63MeH/R67MeH with Cu(II)
EMD-28758	2.0	40.1	calcitonin receptor in complex with Gs and pramlintide analogue peptide San45
EMD-51521	1.99	40.0	glucose/xylose isomerase from *Streptomyces rubiginosus* with cobalt ions in the active site
EMD-14705	1.77	40.0	human apoferritin obtained from ssDNA coated grid
EMD-29664	1.98	45.8	wild-type GAPDH
EMD-28269	1.68	60.0	mouse apoferritin heavy chain without zinc determined using single-particle cryo-EM with Apollo camera
EMD-27833	1.98	52.5	helical arch of BIRC6 (from local refinement 1)
EMD-60915	1.51	50.0	mouse heavy-chain apoferritin
EMD-61726	1.78	50.0	ferritin variant R63BrThA/E67BrThA
EMD-17520	1.8	70.0	CAK in complex with inhibitor ICEC0943

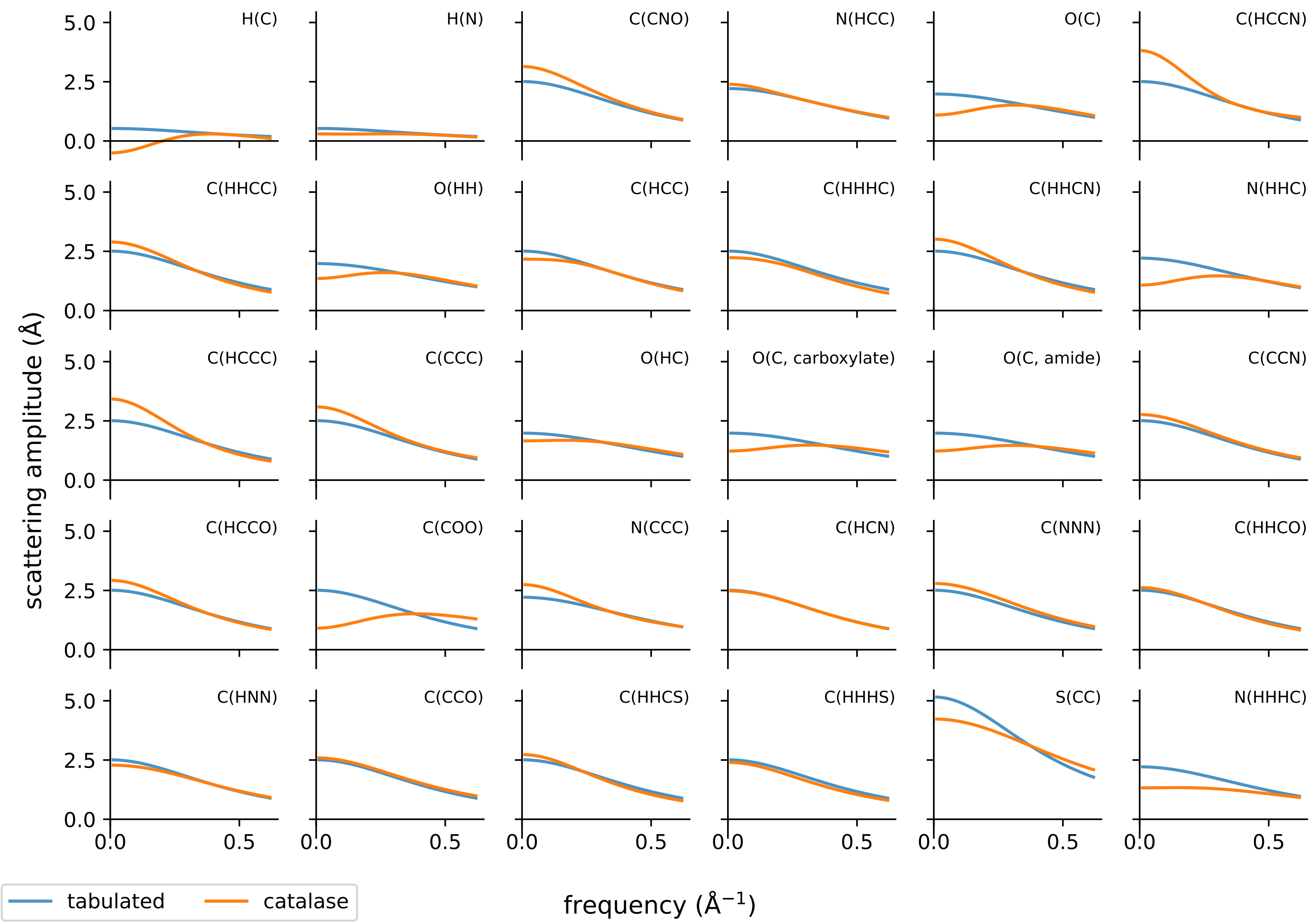

Fig. S1. Tabulated scattering factors (blue) and scattering factors determined from the catalase training set (orange) for the 30 most common atom types in the catalase training set.

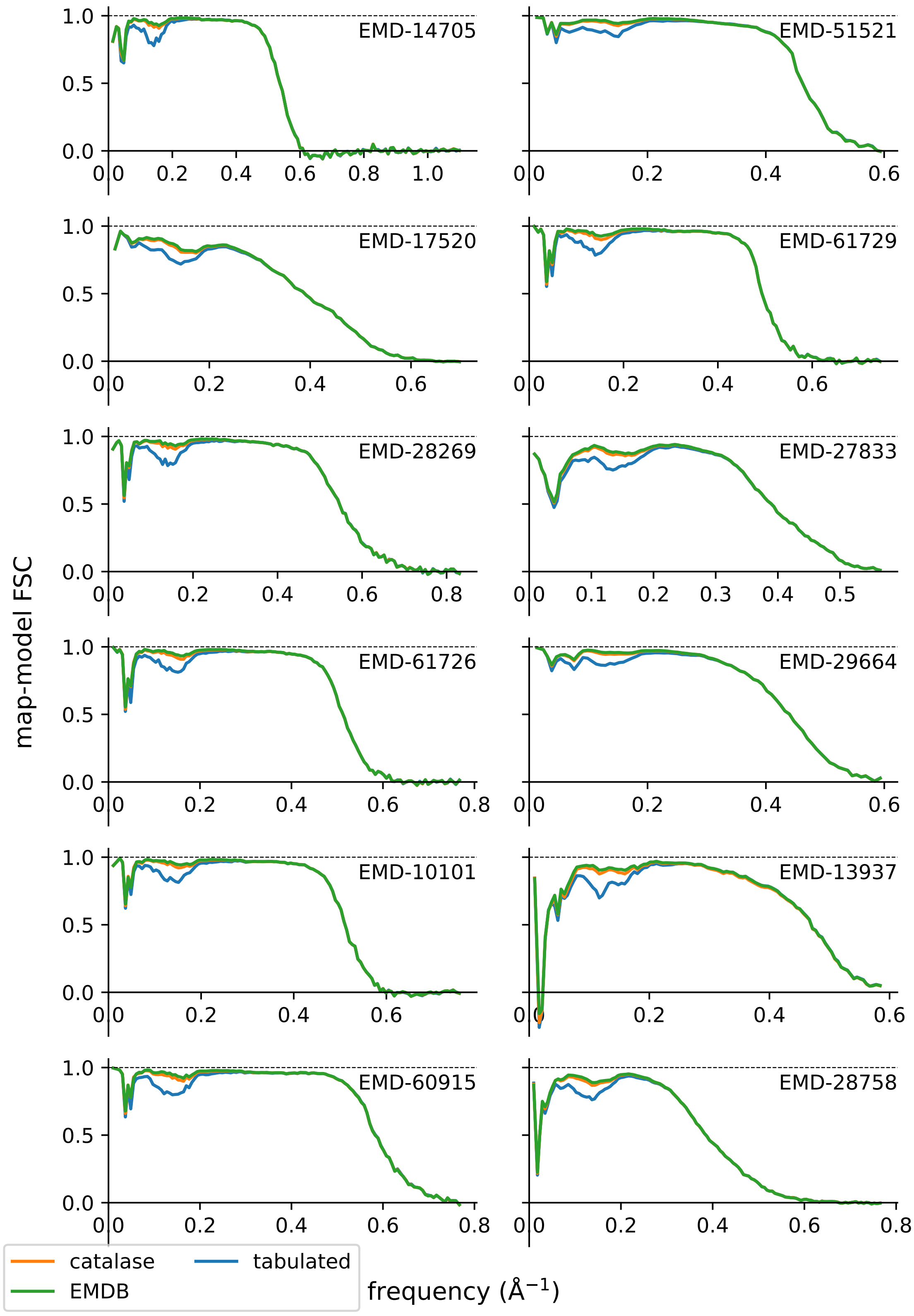

Fig. S2. Map-model FSC for structures in the test set, for scattering amplitudes calculated using different sets of scattering factors.

Alexander Shtyrov, Hugh Wilson, Daria Slowik, Keitaro Yamashita, Jade Li, Marcin Wojdyr, Shaoxia Chen, Greg McMullan, Jude Short, Christopher J. Russo, Richard Henderson and Garib N. Murshudov

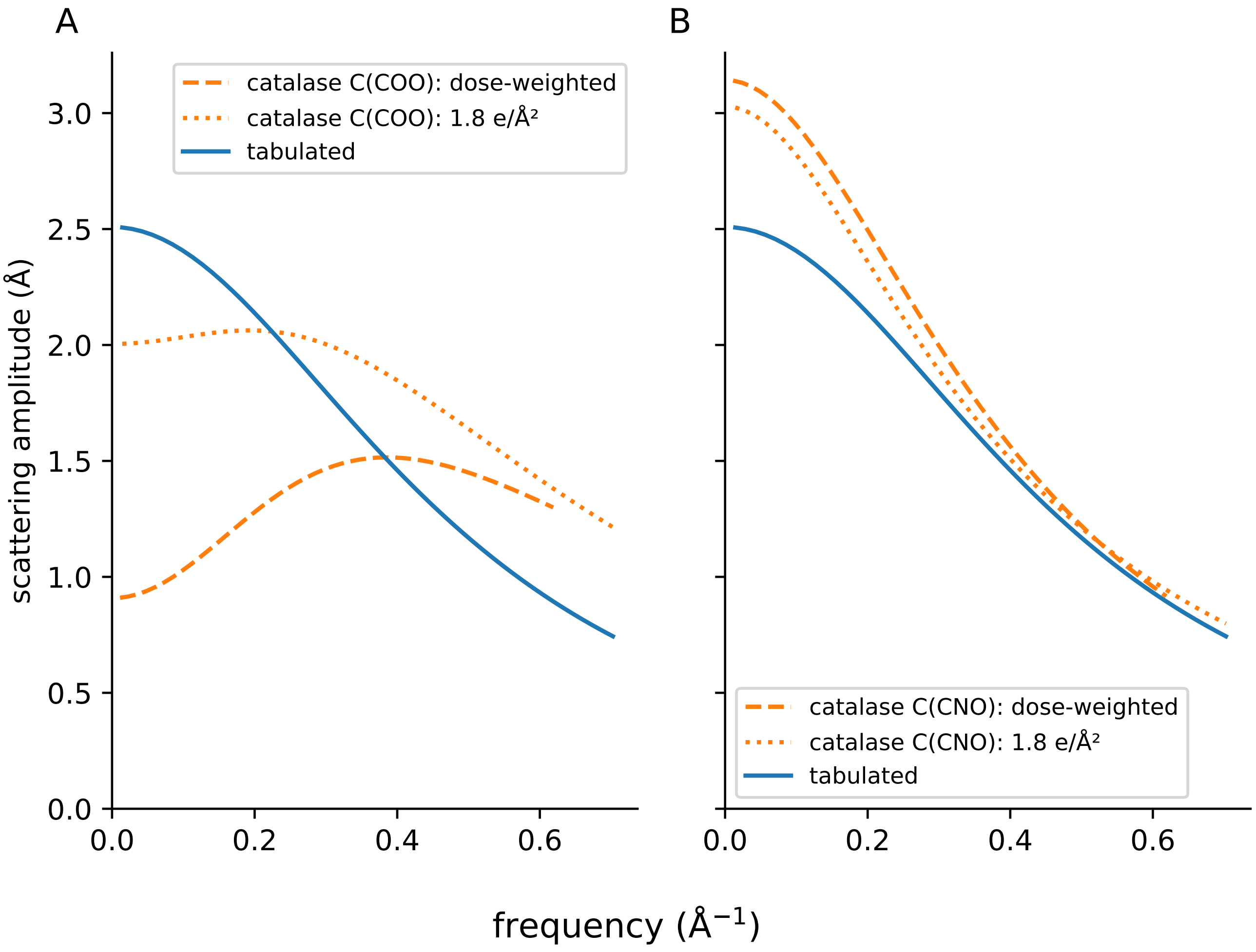

Fig. S3. Empirical scattering factors calculated from the catalase training set at different fluences. Each panel shows the scattering factor for the indicated carbon atom type, calculated either from the *RELION* dose-weighted map or data only from the first movie frame, corresponding to a fluence of 1.8 e$Å^{-2}$. The tabulated scattering factor for carbon is also shown for reference.

References

1. H Kohl, L Reimer, *Transmission Electron Microscopy: Physics of Image Formation.* (Springer, New York), 5th edition, pp. 141–194 (2008).
2. PJ Brown, AG Fox, EN Maslen, MA O'Keefe, BTM Willis, Intensity of diffracted intensities in *International Tables for Crystallography*, ed. E Prince. (John Wiley & Sons), pp. 554–595 (2006).
3. C Colliex, et al., Electron diffraction in *International Tables for Crystallography*, ed. E Prince. (John Wiley & Sons), pp. 259–429 (2006).
4. GN Murshudov, et al., REFMAC5 for the refinement of macromolecular crystal structures. *Acta Crystallogr. Sect. D Biol. Crystallogr.* **67**, 355–367 (2011).
5. K Yamashita, CM Palmer, T Burnley, GN Murshudov, Cryo-EM single-particle structure refinement and map calculation using Servalcat. *Acta Crystallogr. Sect. D Struct. Biol.* **77**, 1282–1291 (2021).
6. C Bishop, *Pattern Recognition and Machine Learning.* (Springer, New York), pp. 291–323 (2006).
7. RH Byrd, P Lu, J Nocedal, C Zhu, A limited memory algorithm for bound constrained optimization. *SIAM J. on Sci. Comput.* **16**, 1190–1208 (1995).
8. N Friel, AN Pettitt, Marginal likelihood estimation via power posteriors. *J. Royal Stat. Soc. Ser. B: Stat. Methodol.* **70**, 589–607 (2008).
9. RJ Read, C Millán, AJ McCoy, TC Terwilliger, Likelihood-based signal and noise analysis for docking of models into cryo-EM maps. *Acta Crystallogr. Sect. D Struct. Biol.* **79**, 271–280 (2023).
10. RC Masmaliyeva, GN Murshudov, Analysis and validation of macromolecular B values. *Acta Crystallogr. Sect. D Struct. Biol.* **75**, 505–518 (2019).
11. Y Zhang, et al., Single-particle cryo-EM: alternative schemes to improve dose efficiency. *J. Synchrotron Radiat.* **28**, 1343–1356 (2021).
